# Identifying biomarkers distinguishing sepsis after trauma from trauma-induced SIRS based on metabolomics data: a retrospective study

**DOI:** 10.1038/s41598-025-94701-y

**Published:** 2025-04-21

**Authors:** Yi Gou, Jing-jing Liu, Jun-fei Zhang, Wan-peng Yang, Jian-Zhong Yang, Ke Feng

**Affiliations:** 1 Department of Emergency Medical, General Hospital of Ningxia Medical University, Yinchuan, 750003 Ningxia China; 2 Department of Emergency Medical, The First Affiliated Hospital of Xinjiang Medical University, Urumqi, 830011 China

**Keywords:** Sepsis, SIRS, Trauma, Biomarkers, Metabolomics, Trauma, Diagnostic markers

## Abstract

Sepsis after trauma and trauma-induced SIRS have similar symptoms, making their differentiation challenging. Therefore, biomarkers are needed to differentiate between sepsis after trauma and trauma-induced SIRS. We hypothesized that sepsis following trauma induces distinct alterations in blood metabolism compared to trauma-induced SIRS and sought to identify metabolite biomarkers in blood that could differentiate between the two. In this retrospective study, the existing blood metabolomics data from 60 patients without trauma-induced SIRS, 40 patients with trauma-induced SIRS, and 50 non-trauma control cases were analyzed. Among 40 traumatic patients with SIRS, 16 developed sepsis (SDS group), 24 did not develop sepsis (SDDS group) within the subsequent two-week period after trauma. A pairwise comparison between SDS group and SDDS group was used to screen the differential metabolites as biomarkers distinguishing sepsis after trauma from trauma-induced SIRS. Using partial least‑squares discriminant analysis, we demonstrated that SDS group was metabolically distinct from the SDDS group. A total of 37 differential metabolites were found between SDS group and SDDS group. We selected 5 most significantly different metabolites between SDS and SDDS groups as biomarkers to discriminate sepsis after trauma from trauma-induced SIRS, which were 7-alpha-carboxy-17-alpha-carboxyethylandrostan lactone phenyl ester, docosatrienoic acid, SM 8:1;2O/26:1, SM 34:2;2O, and N1-[1-(3-isopropenylphenyl)-1-methylethyl]-3-oxobutanamide. Our study has identified the potential of these biomarkers for differentiating sepsis after trauma from trauma-induced SIRS. This not only provides a new approach for the early diagnosis of sepsis after trauma but also lays a solid foundation for further research based on targeted metabolomics, which may lead to the development of more effective treatment strategies in the future.

## Introduction

Trauma burden is one of the leading causes of loss of young human lives and economic loss in low-and middle-income countries^1^. Trauma-related fatalities display a bimodal distribution, with a majority occurring in the initial days following the injury, often due to severe head injury or uncontrollable bleeding^2^. Patients who, with the help of medical and surgical interventions, survive the initial trauma often remain critically ill and are confronted with an increased risk of inflammatory complications^3^. Due to the early release of Damage-Associated Molecular Patterns (DAMPs), severe trauma induces systemic inflammatory response syndrome (SIRS). Subsequently, it leads to a compensatory and persistent anti-inflammatory response. This sequence of events increases the susceptibility to sepsis after trauma, and also raises the morbidity and mortality rates^3–5^. Studies have shown that the prevalence of sepsis in patients with critical trauma in the ICU was about 30%^6^. The occurrence of sepsis is a major concern in trauma patients who have survived initial resuscitation^7^, and it accounts for 10% of trauma-related deaths^8^. Early treatment and detection of sepsis are the key to reducing mortality and cost of care^9^, but it is difficult to detect sepsis after trauma in the early stage, especially for patients with trauma-induced SIRS. In the absence of an unambiguous definition of sepsis and highly accurate diagnostic tools, physicians rely on their own clinical skill set and experience to diagnose sepsis^10^. Clinicians make an ‘educated guess’ that a patient likely has an infection based on their history, classic signs (e.g. pyrexia, tachycardia), examination, and available laboratory, microbiology, and/or radiology results^11^. Blood culture is the gold standard for diagnosing pathogens, but it is time-consuming and successful in less than half of sepsis cases^8,12^. SIRS and sepsis can be considered clinical phenotypes of the host response’s failure to contain local tissue inflammation after sterile and infectious tissue injury, respectively^13^. Clinical symptoms of post-traumatic SIRS include fever, increased heart rate, and shock, which also occur during sepsis^3^. It is reported that ~ 60% of physicians’ initial diagnoses of sepsis in ICU were ultimately classified as having a non-infectious SIRS^10^. So, deciphering sepsis after trauma from non-infective SIRS induced by trauma remains a challenging task^14^. Therefore, identifying biomarkers distinguishing sepsis after trauma from trauma-induced SIRS is key to early diagnosis and treatment of sepsis after trauma. Praveen Papareddy et al.^14^ employed Matrix-Assisted Laser Desorption/Ionization and multiplex antibody arrays to identify biomarkers distinguishing sepsis from trauma-induced sterile inflammation. While it is regrettable that patients in the sepsis group were patients of community-acquired sepsis rather than sepsis after trauma. Metabolism is a systems biology approach for the identification and quantification of global metabolites in biological samples^15^. Metabolomics provides metabolite information in biochemical processes, abnormal metabolites can be used as potential biomarkers for diagnosis, and treatment^16^. Xiaolin Zhang et al. measured plasma concentration of kynurenine using untargeted metabolomics, and found kynurenine could be a robust biomarker for ST-acute myocardial infarction prognosis^17^. Based on metabolomic analysis, Yang Li screened nine metabolites as potential characteristic biomarkers for the diagnosis of sepsis (AUC: 0.782 to 0.941)^18^. In clinical metabolomics, plasma, serum, and urine have been widely used as sample sources to discover disease markers^15^. We hypothesized that sepsis after trauma leads to different changes in blood metabolism from trauma-induced SIRS and searched for metabolite biomarkers in blood to distinguish between the two conditions. So, we retrospectively analyzed the existing blood metabolomics data from 100 patients with severe trauma and 50 non-trauma control. Among 40 traumatic patients with SIRS, 16 developed sepsis (SDS group), 24 did not develop sepsis (SDDS group). A pairwise comparison between SDS group and SDDS group was used to screen the differential metabolites as biomarkers distinguishing sepsis after trauma from trauma-induced SIRS. It was expected to provide clues distinguishing sepsis after trauma from trauma-induced SIRS.

## Methods

### Study design and patient selection

This study retrospectively analyzed the existing blood metabolomics data from 100 patients with severe trauma and 50 healthy controls. These plasma samples were obtained from March 2022 to November 2023 in the General Hospital of Ning Xia Medical University. Plasma samples of severe trauma patients who visited our hospital within 24 h after injury were obtained within the first hour of patient admission. 40 patients with trauma-induced SIRS from the 100 severe trauma patients were classified as SIRS group, the rest of the trauma patients without SIRS were classified as Non-SIRS group. Among SIRS group, 16 SIRS patients developed sepsis (SDS group), 24 SIRS patients did not develop sepsis (SDDS group). The time frame from trauma admission to sepsis diagnosis was 1 ~ 10 day [(5.3 ± 2.3) day]. A pairwise comparison between SDS group and SDDS group was used to screen the differential metabolites as biomarkers distinguishing sepsis after trauma from trauma-induced SIRS. This study was by the ethical standards outlined in the Helsinki Declaration of 1964 and its later amendments. The study protocol was approved by the Medical Ethics Committee of General Hospital of Ningxia Medical University. The need for obtaining informed consent was waived by the Medical Ethics Committee of General Hospital of Ningxia Medical University, because the study used existing metabolomics data. The diagnostic criteria of SIRS were presented in (Table 1). Due to severe trauma-induced injury, heart rate, and respiratory rate were greatly affected, so we considered white blood cell count > 12 000/mm^3^ or < 4000/mm^3^ or > 10% immature bands as necessary criteria of trauma-induced SIRS. We finally screened out 40 patients with trauma-induced SIRS. Sepsis is identified using the Third International Consensus Definitions^19^.

**Table 1:** SIRS.

Two or more of:
Heart rate > 90/min
Temperature > 38 °C or < 36 °C
Respiratory rate > 20/min or PaCO_2_ < 32 mmHg(4.3 kPa)
∗White blood cell count > 12 000/mm^3^ or < 4000/mm^3^ or > 10% immature bands

∗Necessary criteria.

### Data collection

Clinical data were collected on the first day of admission to the Emergency Department, including age, sex, co‑morbidities, injury severity score (ISS), Glasgow Coma scale (GCS), temperature (T), white blood cell count (WBC), hemoglobin (HGB), albumin (Alb), platelet (PLT), invasive mechanical ventilation (MV), open wound, and injury mechanism.

### Statistical analysis

When comparing clinical characteristics between the two groups, continuous variables were expressed as mean ± standard deviation for normally distributed data and as median (interquartile range, 25–75%) for non-normally distributed data,, and categorical variables are reported as numbers (percentages). Independent sample t-tests were used for normally distributed data, while the Mann-Whitney test was used for non-normally distributed data. Pearson’s χ2 test or Fisher’s exact test were used to compare categorical variables. Significance was defined by *p* < 0.05. For all analyses, a two-tailed p-value < 0.05 was considered statistically significant. Statistical analysis was performed using IBM SPSS version 26.0 (IBM Corp., Armonk, NY, USA). Partial least-squares discriminant analysis (PLS-DA) was performed using MetaboAnalyst 6.0. The differential metabolites were identified based on the criteria of variable importance in projection (VIP) score > 1.0 and P value < 0.05. Area under the curve (AUC) analysis and a heatmap using Euclidean and T-test were also performed using MetaboAnalyst 6.0.

## Results

### Patients’ characteristics

50 healthy controls, 60 patients without trauma-induced SIRS, and 40 patients with trauma-induced SIRS were included. Table 2 showed the comparison of the clinical characteristics between the control group and the Non-SIRS group. The comparison of the clinical characteristics for the control group and the SIRS group could be found in (Table 3). As for the comparison between the Non-SIRS group and the SIRS group in terms of their clinical characteristics, it was presented in (Table 4). The comparison of the clinical characteristics of SDS group and SDDS group is presented in (Table 5). There was no statistically significant difference in age, male, ISS, co‑morbidities, and injury mechanisms between the two groups.

**Table 2:** Baseline characteristics of control compared with non‑sirs.

Characteristics	Non-SIRS (*n* = 60)	Control (*n* = 50)	t/Z/χ2	*P*-value
Male (n, %)	46(76.7)	39(78.0)	0.03	0.868
Age	52.7 ± 15.8	43.5 ± 15.2	−2.30	0.002
Laboratory findings				
WBC(×10^9^/L)	16.2(9.9–20.5)	6.8 (5.7–8.3)	9.91	<0.001
HGB (g/L)	119.5 (97.5-135.8)	159.0 (148.5-166.5)	−9.73	<0.001
PLT (×10^9^/L)	184.8 ± 70.0	248.1 ± 74.4	−4.56	<0.001
ALB (g/L)	33.4 (26.4–38.2)	46.1 (44.5–48.8)	−9.50	<0.001
ISS	23.0 (17.0–34.0)	NA		
GCS	13.0 (6.0–15.0)	NA		
Open wound (n, %)	37 (61.7)	NA		
Invasive MV (n, %)	38 (63.3)	NA		
Injury mechanism (n, %)	*N* = 60	NA		
Traffic accident (n, %)	43 (71.7)			
Fall	14 (23.3)			
Others	3 (5.0)			

*SIRS* systemic inflammatory response syndrome, *WBC* white blood cell count, *HGB* hemoglobin, *PLT* platelet, *Alb* albumin, *ISS* injury severity score, *GCS* glasgow coma scale, *MV* mechanical ventilation.

**Table 3:** Baseline characteristics of control group compared with SIRS group.

Characteristics	SIRS (*n* = 40)	Control (*n* = 50)	t/Z/χ2	*P*-value
Male (n, %)	33(82.5)	39(78.0)	0.28	0.596
Age	42.9 ± 16.5	43.5 ± 15.2	0.18	0.858
Laboratory findings				
WBC(×10^9^/L)	19.2(16.3–26.4)	6.8 (5.7–8.3)	−13.63	<0.001
HGB (g/L)	124.0 (105.3–139.0)	159.0 (148.5-166.5)	6.98	<0.001
PLT (×10^9^/L)	225.1 ± 73.4	248.1 ± 74.4	1.47	0.145
ALB (g/L)	34.4 (29.2–41.1)	46.1 (44.5–48.8)	8.46	<0.001
ISS	26.0 (17.0–34.0)	NA		
GCS	13.0 (6.0–15.0)	NA		
Open wound (n, %)	28 (70.0)	NA		
Invasive MV (n, %)	16 (40.0)	NA		
Injury mechanism (n, %)	*N* = 40	NA		
Traffic accident (n, %)	31 (77.5)			
Fall	7 (17.5)			
Others	2 (5.0)			

*SIRS* systemic inflammatory response syndrome, *WBC* white blood cell count, *HGB* hemoglobin, *PLT* platelet, *Alb* albumin, *ISS* injury severity score, *GCS* glasgow coma scale, *MV* mechanical ventilation.

**Table 4:** A comparison of clinical characteristics of Non-SIRS and SIRS groups.

Characteristics	Non-SIRS (*n* = 60)	SIRS (*n* = 40)	χ2/t/Z	*P*-value
Age	52.7 ± 15.8	42.9 ± 16.5	2.04	0.003
Male (n, %)	46 (76.7)	33 (82.5)	0.49	0.483
Co‑morbidities (n, %)	23 (38.3)	10 (25.0)	1.930	0.165
ISS	26.3 ± 10.5	26.7 ± 10.8	−0.20	0.845
GCS	10.6 ± 4.5	10.8 ± 4.7	−0.12	0.902
T (℃)	36.8 ± 0.5	36.6 ± 0.5	1.63	0.106
WBC (103/mm^3^)	16.2 ± 6.9	21.4 ± 6.5	−3.79	<0.001
HGB (g/L)	117.3 ± 26.1	120.5 ± 29.5	−0.55	0.587
Alb (g/ml)	32.3 ± 8.3	33.8 ± 9.0	−0.84	0.405
PLT (×109/L)	184.8 ± 70.0	225.1 ± 73.6	−2.76	0.007
Invasive MV (n, %)	38 (63.3)	16 (40.0)	23.04	<0.001
Open wound (n, %)	37 (61.7)	28 (70.0)	0.73	0.392
Injury mechanism (n, %)			0.50	0.779
Traffic accident	43 (71.7)	31 (77.5)		
Fall	14 (23.3)	7 (17.5)		
Others	3(5.0)	2 (5.0)		

*SIRS* systemic inflammatory response syndrome, *ISS* injury severity score, *GCS* glasgow coma scale, *T* temperature, *WBC* white blood cell count, *HGB* hemoglobin, *Alb* albumin, *PLT* platelet, *MV* mechanical ventilation.

**Table 5:** A comparison of clinical characteristics of SDS group and SDDS group.

Characteristics	SDS (*n* = 16)	SDDS (*n* = 24)	χ2/t/Z	*P*-value
Age	40.8 ± 15.8	44.3 ± 17.2	−0.67	0.508
Male (n, %)	15 (93.8)	18 (75.0)	2.34	0.126
Co‑morbidities (n, %)	3 (18.8)	7 (29.2)	0.56	0.456
ISS	29.1 ± 10.3	25.0 ± 11.0	1.18	0.247
GCS	10.3 ± 5.2	11.0 ± 4.4	−0.48	0.637
T (℃)	36.7 ± 0.5	36.6 ± 0.4	0.99	0.330
WBC (103/mm^3^)	21.7 ± 5.5	21.1 ± 7.1	0.28	0.781
HGB (g/L)	116.3 ± 33.1	123.3 ± 27.2	−0.72	0.473
Alb (g/ml)	32.2 ± 10.1	34.9 ± 8.2	−0.91	0.366
PLT (×109/L)	227.9 ± 71.6	223.2 ± 76.4	0.20	0.846
Invasive MV (n, %)	8 (50.0)	8 (33.3)	1.11	0.290
Open wound (n, %)	12 (75.0)	16 (66.7)	0.32	0.573
Injury mechanism (n, %)			0.13	0.938
Traffic accident	12 (75.0)	19 (79.2)		
Fall	3 (18.8)	4 (16.7)		
Others	1(6.3)	1 (4.2)		

*ISS* injury severity score, *GCS* glasgow coma scale, *T* temperature, *WBC* white blood cell count, *HGB* hemoglobin, *Alb* albumin, *PLT* platelet, *MV* mechanical ventilation.

### PLS-DA analyzing to discriminate two groups

**Fig. 1:**
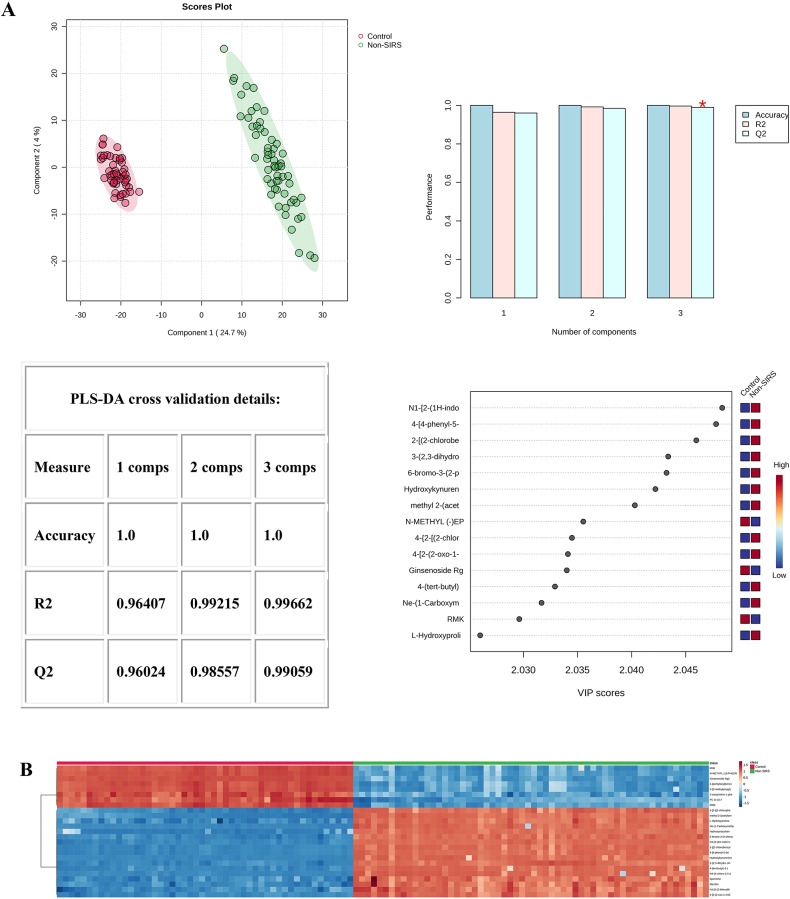

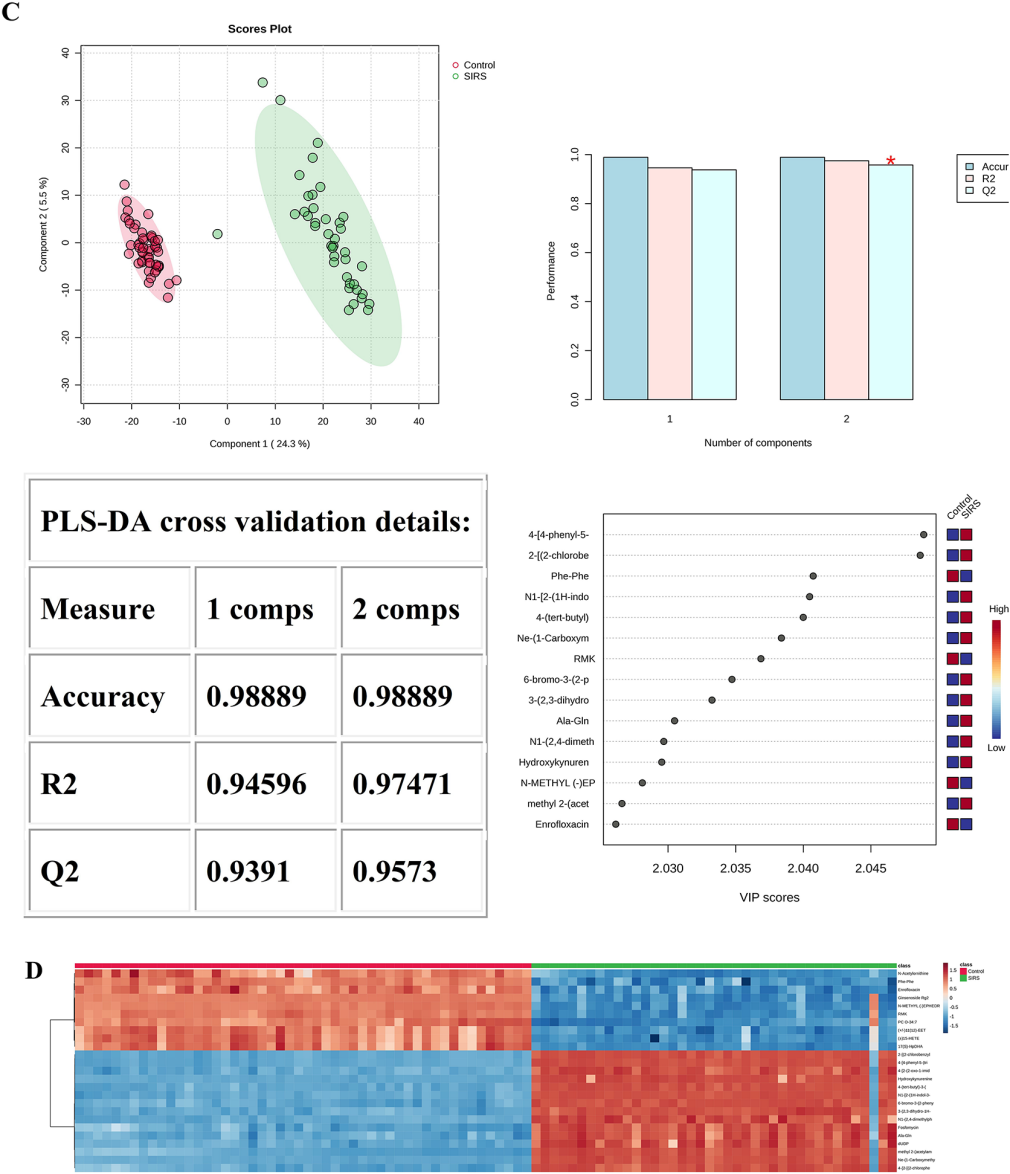

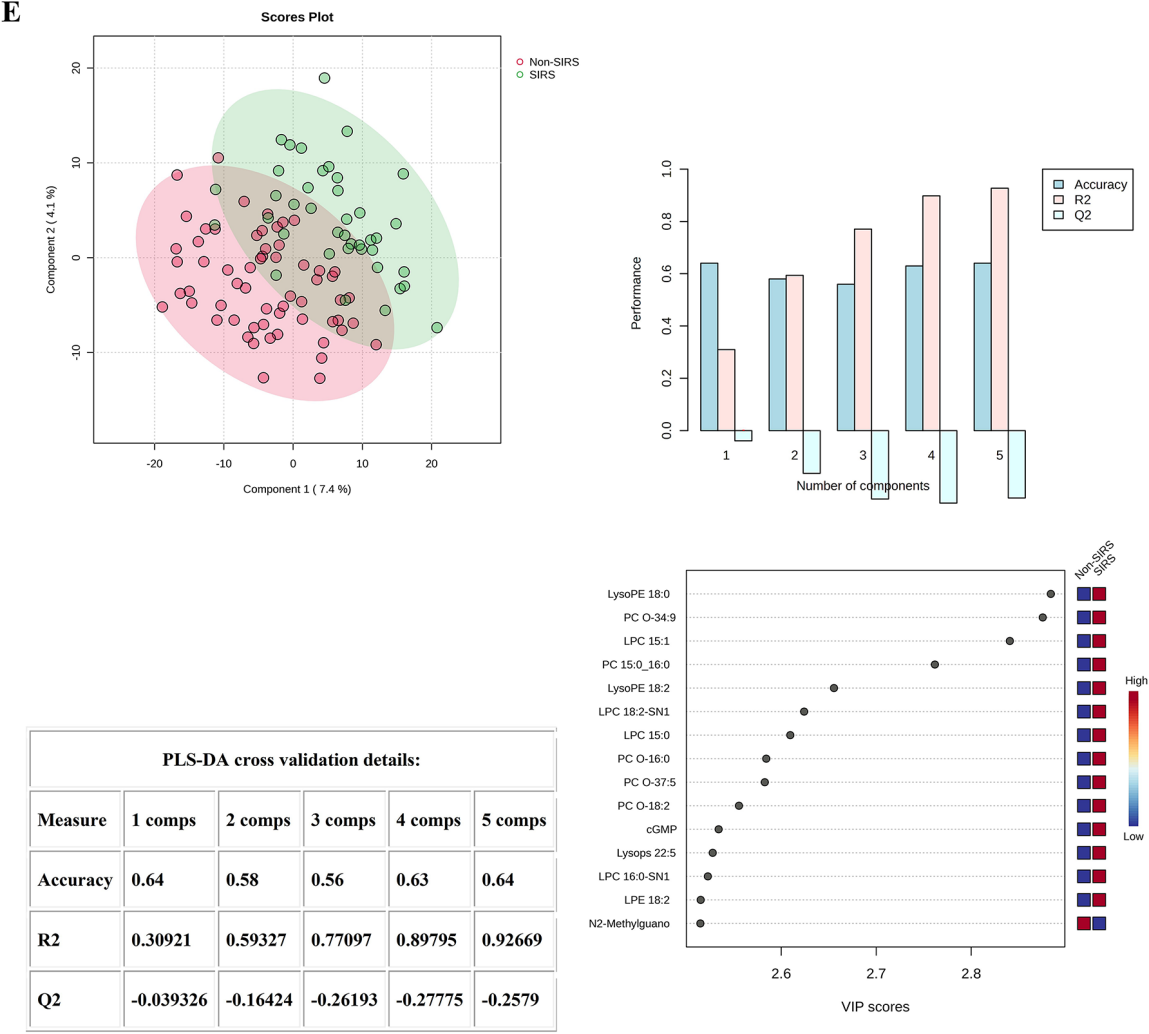

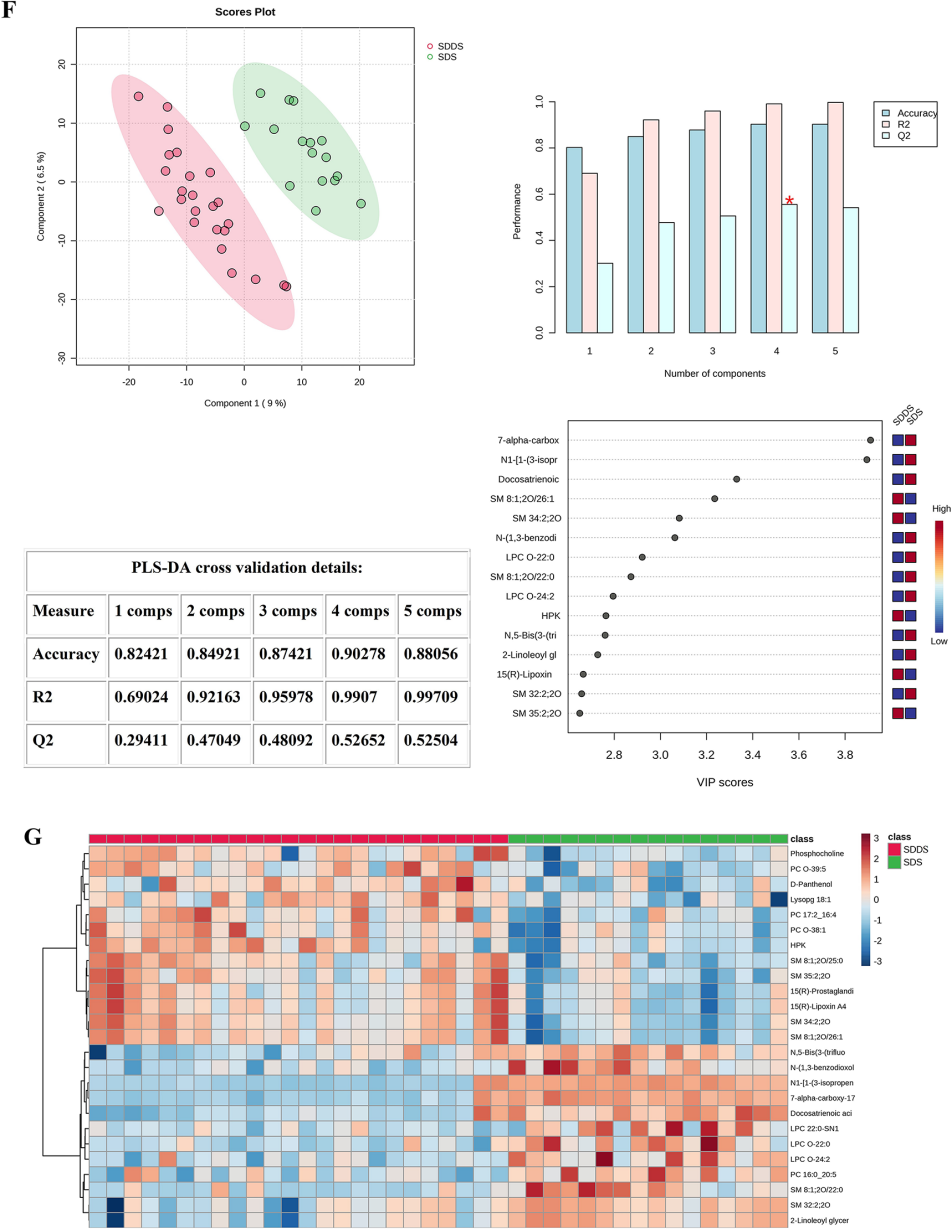
Statistical analysis of the data obtained for 50 Control (50), Non-SIRS (60), and SIRS (40) groups. (**A**) PLS‑DA for Control and Non-SIRS groups: 2D score plot for the discrimination of Control groupand Non-SIRS group, important metabolites discriminating the two groups. (**B**) Hierarchical heatmap for top‑25 discriminating metabolites between Control and Non-SIRS groups (red bar: Control group, green bar: Non-SIRS group). (**C**) PLS‑DA for Control and SIRS groups: 2D score plot for the discrimination of Control group and SIRS group, important metabolites discriminating the two groups. (**D**) Hierarchical heatmap for top‑25 discriminating metabolites between Control and SIRS groups (red bar: Control group, green bar: SIRS group). (**E**) PLS‑DA for Non-SIRS and SIRS groups: 2D score plot for the discrimination of Non-SIRS group and SIRS group, important metabolites discriminating the two groups. (**F**) PLS‑DA for SDS and SDDS groups: 2D score plot for the discrimination of SDS group and SDDS group, important metabolites discriminating the two groups. (**G**) Hierarchical heatmap for top‑25 discriminating metabolites between SDS and SDDS groups (red bar: SDDS group, green bar: SDS group). VIP score: the metabolites are responsible for discrimination between sepsis after trauma and trauma-induced SIRS. Metabolites with high VIP scores are more important in class separation.

When analyzing the Control and Non-SIRS groups using PLS-DA, 2-dimensional score plotting showed that the two groups were distinguished by metabolic profiles (Fig. 1A), and we chose 3 components which were achieved by cross-validation method of PLS-DA with R^2^ = 1.00, Q^2^ = 0.99, and accuracy of 1.0. A high R^2^ value close to 1 indicates an excellent fit of the model to the data, and a high Q^2^ value suggests good predictive ability of the model. The top 15 metabolites based on VIP score were shown (Fig. 1A). Similarly, 2-dimensional score plotting showed that the Control and SIRS groups were distinguished by metabolic profiles (Fig. 1C), we chose 2 components which were achieved by cross-validation method of PLS-DA with R^2^ = 0.97, Q^2^ = 0.96, and accuracy of 0.99. These values also imply a relatively good performance of the model in differentiating the two groups. The top 15 metabolites based on VIP score were shown (Fig. 1C). By utilizing Euclidean distance and T-test, a heatmap was constructed to provide an intuitive visualization of the discriminant metabolites, which were observed between the Control and Non-SIRS groups (Fig. 1B) as well as between the Control and SIRS groups (Fig. 1D). Notably, some important metabolites for distinguishing the Non-SIRS group from the Control group and for distinguishing the SIRS group from the Control group are overlapping. This overlap may suggest that it is difficult to distinguish the SIRS group from the Non-SIRS group by using these metabolites alone. As shown in (Fig. 1E), the Non-SIRS and SIRS groups were not distinguished by metabolic profiles. The value of Q^2^ was less than 0. In general, a Q^2^ value below 0 implies that the model has a poor predictive ability and is likely to be over-fitting.

2-dimensional score plotting showed that SDS group and SDDS group were distinguished by metabolic profiles, and we chose 4 components which were achieved by cross-validation method of PLS-DA with R^2^ = 0.99, Q^2^ = 0.53, and accuracy of 0.9 (Fig. 1F). The top 15 metabolites based on VIP score were shown (Fig. 1F). Heatmap showed the intuitive visualization of discriminant metabolites between the SDS and SDDS groups (Fig. 1G).

### Screening of differential metabolites

This study used a pairwise comparison to screen the differential metabolites. The differential metabolites were identified based on the criteria of VIP value > 1.0 and P value < 0.05. A total of 521 differential metabolites were found between Control group and Non-SIRS group, the top 15 significantly different metabolites are shown in (Table 6). 511 differential metabolites were found between Control and SIRS group, the top 15 significantly different metabolites are shown in (Table 7). Among these significantly different metabolites, 11 metabolites overlapped between the comparison of the Control group vs. the Non-SIRS group and the Control group vs. the SIRS group (Tables 6 and 7). This overlap may provide insights into the common metabolic pathways affected by trauma, regardless of whether SIRS develops or not. 502 metabolites with VIP value > 1.0 were found between the Non-SIRS and SIRS groups; however, after performing a t-test, no metabolites with significant differences were found between the two groups. A total of 37 differential metabolites were found between the SDS and the SDDS. The top 15 significantly different metabolites are shown in (Table 8).

**Table 6:** Top 15 significantly different metabolites between control and non-SIRS groups.

Metabolites	t.stat	*P*.value	VIP
N1-[2-(1 H-indol-3-yl)ethyl]-3,4-dichlorobenzene-1-sulfonamide	−156.32	4.30E-129	2.0484
4-[4-phenyl-5-(trifluoromethyl)-3-thienyl]thiomorpholine-3,5-dione	−147.35	2.47E-126	2.0478
2-[(2-chlorobenzyl)sulfanyl]-4,6-dimethylnicotinonitrile	−126.27	3.92E-119	2.046
3-(2,3-dihydro-1 H-indol-1-yl)-2-[(2-furylmethyl)sulfonyl]acrylonitrile	−107.54	1.15E-111	2.0434
6-bromo-3-(2-phenylethanhydrazonoyl)-2 H-chromen-2-one	−106.71	2.66E-111	2.0432
Hydroxykynurenine	−101.39	6.27E-109	2.0422
Methyl 2-(acetylamino)-4-amino-4-oxobutanoate	−93.365	4.20E-105	2.0403
N-Methyl (-)Ephedrine	79.383	1.33E-97	2.0355
4-{2-[(2-chlorophenoxy)methyl]-1,3-thiazol-4-yl}benzonitrile	−77.064	3.09E-96	2.0345
4-[2-(2-oxo-1-imidazolidinyl)ethyl]-1lambda ~ 6~,4-thiazinane-1,1-dione	−76.278	9.18E-96	2.0341
Ginsenoside Rg2	76.067	1.23E-95	2.034
4-(tert-butyl)-3-(4-chlorophenethyl)-2,3-dihydro-1,3-thiazole-2-thione	−73.931	2.52E-94	2.0329
Ne-(1-Carboxymethyl)-L-lysine	−71.701	6.45E-93	2.0317
RMK	68.405	9.32E-91	2.0296
L-Hydroxyproline	−63.555	2.17E-87	2.026

**Table 7:** Top 15 significantly different metabolites between control and SIRS groups.

Metabolites	t.stat	*P*.value	VIP
4-[4-phenyl-5-(trifluoromethyl)-3-thienyl]thiomorpholine-3,5-dione	−46.185	1.76E-63	2.0489
2-[(2-chlorobenzyl)sulfanyl]-4,6-dimethylnicotinonitrile	−46.037	2.31E-63	2.0486
Phe-Phe	42.115	4.17E-60	2.0407
N1-[2-(1 H-indol-3-yl)ethyl]-3,4-dichlorobenzene-1-sulfonamide	−42.001	5.23E-60	2.0405
4-(tert-butyl)-3-(4-chlorophenethyl)-2,3-dihydro-1,3-thiazole-2-thione	−41.798	7.86E-60	2.04
Ne-(1-Carboxymethyl)-L-lysine	−41.124	3.07E-59	2.0384
RMK	40.519	1.06E-58	2.0369
6-bromo-3-(2-phenylethanhydrazonoyl)-2 H-chromen-2-one	−39.706	5.76E-58	2.0347
3-(2,3-dihydro-1 H-indol-1-yl)-2-[(2-furylmethyl)sulfonyl]acrylonitrile	−39.171	1.78E-57	2.0333
Ala-Gln	−38.221	1.37E-56	2.0305
N1-(2,4-dimethylphenyl)-2-(2,4-dichlorophenoxy)propanamide	−37.959	2.43E-56	2.0297
Hydroxykynurenine	−37.911	2.70E-56	2.0295
N-Methyl (-)Ephedrine	37.455	7.37E-56	2.0281
Methyl 2-(acetylamino)-4-amino-4-oxobutanoate	−36.991	2.07E-55	2.0266
Enrofloxacin	36.853	2.81E-55	2.0261

**Table 8:** Top 15 significantly different metabolites between SDS and SDDS groups.

Metabolites	t.stat	*P*. value	VIP	AUC
7-alpha-carboxy-17-alpha-carboxyethylandrostan lactone phenyl ester	−11.121	1.6414E-13	3.9101	0.961
Docosatrienoic acid	−6.8838	3.5335E-08	3.3304	0.948
SM 8:1;2O/26:1	6.4636	1.3195E-07	3.2352	0.943
SM 34:2;2O	5.866	8.6922E-07	3.0818	0.932
N1-[1-(3-isopropenylphenyl)-1-methylethyl]-3-oxobutanamide	−10.932	2.71E-13	3.8941	0.927
2-Linoleoyl glycerol	−4.7493	0.000028958	2.7285	0.917
SM 32:2;2O	−4.5607	0.000051821	2.6589	0.914
N-(1,3-benzodioxol-5-yl)-2-methyl-5-(piperidinosulfonyl)-3-furamide	−5.7973	1.08E-06	3.0627	0.896
HPK	4.8491	0.000021247	2.764	0.891
N,5-Bis(3-(trifluoromethyl)phenyl)oxazol-2-amine	−4.8395	0.000021889	2.7606	0.888
LPC O-22:0	−5.3217	4.84E-06	2.9214	0.883
LPC O-24:2	−4.939	1.61E-05	2.7953	0.880
Phosphocholine	4.0926	0.00021436	2.4727	0.878
SM 8:1;2O/22:0	−5.1689	7.8252E-06	2.8724	0.878
15(R)-Lipoxin A4	4.5793	4.89E-05	2.6659	0.857

### Biomarkers distinguishing sepsis after trauma from trauma-induced SIRS

**Fig. 2:**
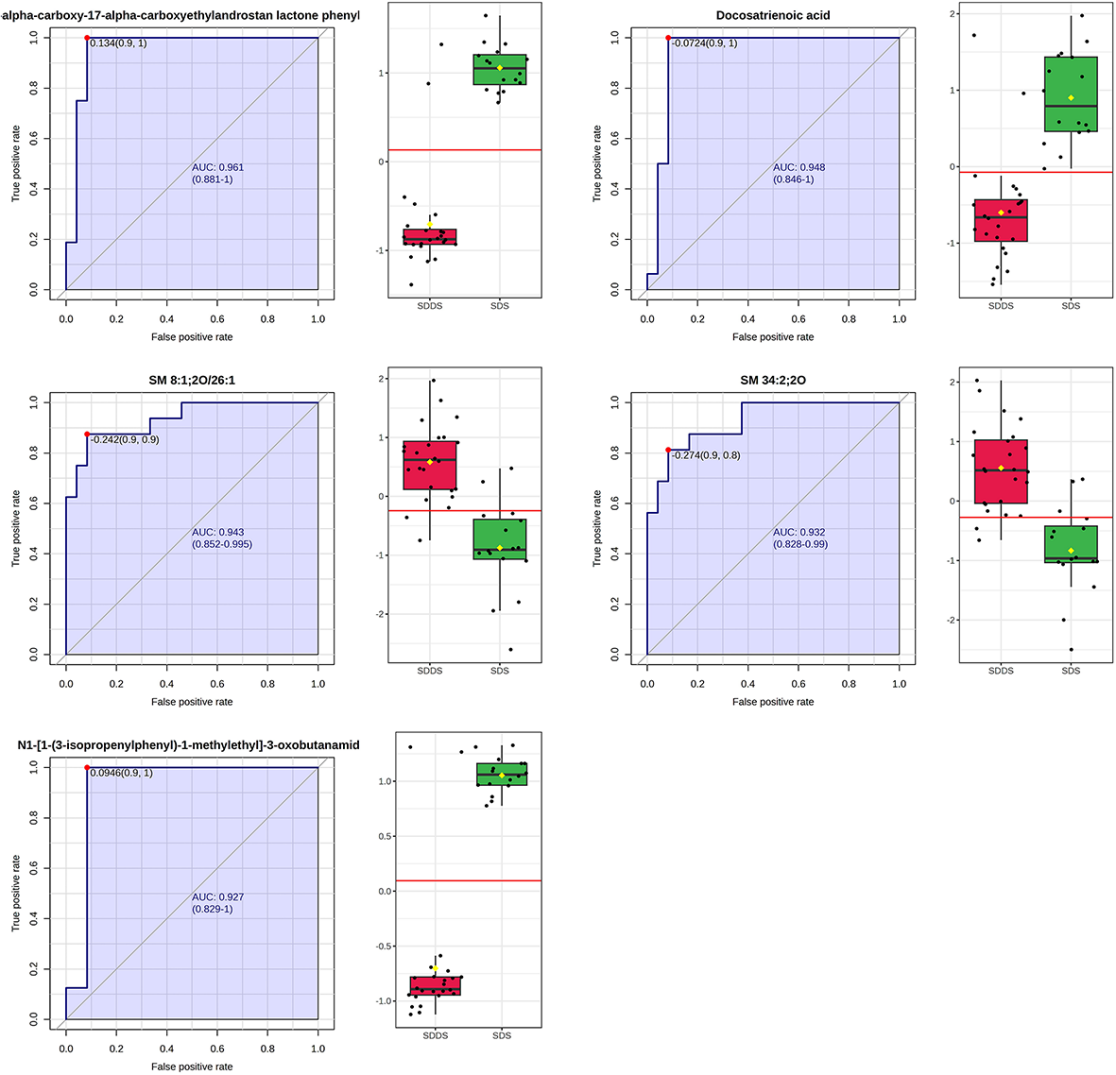
Biomarkers distinguishing sepsis after trauma from trauma-induced SIRS.

We selected 5 most significantly different metabolites between SDS and SDDS groups as biomarkers to discriminate sepsis after trauma from trauma-induced SIRS, which were 7-alpha-carboxy-17-alpha-carboxyethylandrostan lactone phenyl ester, docosatrienoic acid, SM 8:1;2O/26:1, SM 34:2;2O, and N1-[1-(3-isopropenylphenyl)-1-methylethyl]-3-oxobutanamide. Lower concentrations of SM 8:1;2O/26:1 and SM 34:2;2O were observed in the SDS group as compared to the SDDS group, while the concentration of 7-alpha-carboxy-17-alpha-carboxyethylandrostan lactone phenyl ester, docosatrienoic acid, and N1-[1-(3-isopropenylphenyl)-1-methylethyl]-3-oxobutanamide were higher in the SDS group (Fig. 2). Receiver operating characteristic (ROC) curves were used to predict the diagnostic potential of these metabolic biomarkers for Sepsis, AUCs are greater than 0.9, indicating an excellent discriminatory ability (Fig. 2).

## Discussion

Sepsis, which poses a significant global burden, has attracted a large number of studies focusing on its early diagnosis and treatment. Post-traumatic sepsis is a subtype of sepsis. Compared with non-traumatic sepsis, in the early stage of trauma, there exists organ dysfunction and SIRS, which renders it arduous to differentiate between post-traumatic sepsis and trauma-induced SIRS. This situation is highly likely to result in the delayed diagnosis of post-traumatic sepsis or the misuse of antibiotics. Therefore, screening out biomarkers capable of distinguishing between post-traumatic sepsis and trauma-induced SIRS holds great significance for the early diagnosis of post-traumatic sepsis. Nevertheless, there are scant related studies. To address this problem, we employed existing metabolomics data to identify biomarkers distinguishing sepsis after trauma from trauma-induced SIRS. The metabolomics data came from 16 traumatic patients with SIRS, who developed sepsis and 24 traumatic patients with SIRS, who did not develop sepsis. We finally selected 5 most significant metabolites as biomarkers to discriminate sepsis after trauma from trauma-induced SIRS, which were 7-alpha-carboxy-17-alpha-carboxyethylandrostan lactone phenyl ester, docosatrienoic acid, SM 8:1;2O/26:1, SM 34:2;2O, and N1-[1-(3-isopropenylphenyl)-1-methylethyl]-3-oxobutanamide among 37different metabolites. Other potential biomarkers are shown in (Table 8). The top 15 biomarkers are mainly lipid metabolites, including docosatrienoic acid, SM 8:1;2O/26:1, SM 34:2;2O, 2-Linoleoyl glycerol, SM 32:2;2O, LPC O-22:0, LPC O-24:2, phosphocholine, SM 8:1;2O/22:0, and 15(R)-Lipoxin A4. These metabolites may be important biomarkers for distinguishing post-traumatic sepsis from trauma-induced SIRS. Furthermore, it suggests that lipid metabolism, especially the metabolism of lysophosphatidylcholine (LPC) and sphingomyelin (SM), is closely related to the occurrence and development of post-traumatic sepsis. However, the origins of certain metabolites, along with the underlying mechanisms through which they are involved in post-traumatic sepsis, remain elusive. These metabolites include 7-alpha-carboxy-17-alpha-carboxyethylandrostan lactone phenyl ester, N1-[1-(3-isopropenylphenyl)-1-methylethyl]-3-oxobutanamide, N-(1,3-benzodioxol-5-yl)-2-methyl-5-(piperidinosulfonyl)-3-furamide, and N,5-Bis(3-(trifluoromethyl)phenyl)oxazol-2-amine. Consequently, further cell experiments, animal experiments, and clinical studies are imperative to delve deeply into their specific mechanisms and functions in the context of post-traumatic sepsis.

SM, a class of lipid molecules that not only have the ability to form and support structures but also execute a variety of biological function, has been proven to play a crucial role in important physiological processes such as the regulation of cell membrane fluidity, signal transduction, inflammation and apoptosis^20^. In the study by Ha-Yeun Chung et al.^21^, it was reported that acid SM is associated with endothelial stress response in sepsis and systemic inflammation. Espen Melum et al.^22^ found that in mice lacking acid sphingomyelinase, there was a reduction in CD1d - restricted antigen presentation in the thymus, and the selection of invariant natural killer T (iNKT) cells was affected, resulting in a decrease in the level of iNKT cells and resistance to the inflammatory state mediated by iNKT cells. Their findings imply that SM is vital for the development and function of iNKT cells in vivo. In sepsis, SM may affect the functions of immune cells and the process of the inflammatory response. Interestingly, in comparison to the SDDS group, the concentrations of some SMs were higher while those of others were lower in the SDS group (Fig. 1G). This may indicate that the functions of different SMs vary. Moreover, the dynamic regulation of these SMs is highly likely closely associated with the occurrence of post-traumatic sepsis, providing clues for a further understanding of the disease mechanism.

LPC originates from the cleavage of phosphatidylcholine by phospholipase A2, and it can induce the migration of lymphocytes and macrophages, increase the production of pro-inflammatory cytokines, induce oxidative stress, and promote apoptosis, which can aggregate inflammation and promote the development of diseases^23^. In a study, Siping Liang et al.^24^ demonstrated that glucocorticoid-induced tumor necrosis factor receptor-related protein increased the uptake of LPC by macrophages during sepsis and promoted the LPC-induced activation of the NACHT, LRR, and PYD domain-containing protein 3 inflammasome. Similarly, Yang Liao et al.^25^ demonstrated the accumulation of LPC contributed to heightened oxidative stress and inflammation in the organism. In this study, compared with the SDDS group, the concentrations of LPC were lower in the SDS group. Similarly, Trovato FM et al.^26^found that reduced LPC levels are biomarkers of poor prognosis in individuals with acute liver failure, the LPC-ATX-LPA axis appears to modulate innate immune response in acute liver failure. In conclusion, LPC is related to the mechanisms of inflammation regulation and sepsis. This study provides clues for exploring the role of LPC in the mechanism of post-traumatic sepsis. Further multi-omics, multi-center studies and animal experiments are still needed for verification.

However, this study has certain limitations. First, the sample size was relatively small, and all samples were from a single center. A small sample size may not cover all possible influencing factors, limiting the generalizability of the research results. Patients from different regions, ethnic groups, or hospitals may vary, and these differences may affect metabolite levels and research conclusions. Single - center studies may also have selection bias, and the sample characteristics may not represent the broader trauma patient population. Second, this study is a retrospective study that relies on existing metabolomics data. Retrospective studies cannot prospectively control variables, and there may be confounding factors affecting the accuracy of the research results. Third, the study lacks an external validation cohort, and it is impossible to verify the effectiveness and stability of the screened biomarkers in an independent patient group. This has raised certain doubts about the reliability of the research results in clinical applications. Despite these shortcomings, this study still holds great significance. For the first time, through metabolomic methods, it has revealed the metabolic differences between post - traumatic sepsis and trauma - induced SIRS to a certain extent, providing a direction for subsequent research. The screened biomarkers have laid the foundation for further targeted metabolomics - based research. Future studies can expand the sample size, conduct multi - center research, and set up validation cohorts to conduct more in - depth verification and evaluation of these biomarkers. Additionally, by integrating other omics technologies, such as genomics and transcriptomics, it will be possible to explore the pathogenesis of post - traumatic sepsis from multiple levels, providing strong support for the development of more effective diagnostic and treatment strategies.metabolomics.

## Conclusion

In conclusion, despite these limitations, our study has identified the potential of employing metabolic biomarkers for differentiation of sepsis after trauma from trauma-induced SIRS. In particular, 7-alpha-carboxy-17-alpha-carboxyethylandrostan lactone phenyl ester, docosatrienoic acid, SM 8:1;2O/26:1, SM 34:2;2O, and N1-[1-(3-isopropenylphenyl)-1-methylethyl]-3-oxobutanamide demonstrated potential as important markers for distinguishing sepsis after trauma from trauma-induced SIRS. This study has laid the foundation for further research based on targeted metabolomics.

## Data Availability

Data are available upon request from Ke Feng (Email: fengkedoct@163.com).

## Abbreviations

SIRS: Systemic inflammatory response syndrome
DAMPs: Damage-associated molecular patterns
ISS: Injury severity score
GCS: Glasgow coma scale
T: Temperature
WBC: White blood cell count
HGB: Hemoglobin
Alb: Albumin
PLT: Platelet
MV: Mechanical ventilation
VIP: Variable importance in projection
PLS-DA: Partial least-squares discriminant analysis
ROC: Receiver operating characteristic
AUC: Area under the curve
LPC: Lysophosphatidylcholine
SM: Sphingomyelin
iNKT: Invariant natural killer T
